# Removal of Lead (II) Ions from Aqueous Solutions onto Activated Carbon Derived from Waste Biomass

**DOI:** 10.1155/2013/146092

**Published:** 2013-06-18

**Authors:** Murat Erdem, Suat Ucar, Selhan Karagöz, Turgay Tay

**Affiliations:** ^1^Department of Chemistry, Faculty of Science, Anadolu University, 26470 Eskisehir, Turkey; ^2^Chemistry Program, Izmir Vocational School, Dokuz Eylül University, Buca, 35160 Izmir, Turkey; ^3^Department of Chemistry, Faculty of Science, Karabük University, Karabük, Turkey

## Abstract

The removal of lead (II) ions from aqueous solutions was carried out using an activated carbon prepared from a waste biomass. The effects of various parameters such as pH, contact time, initial concentration of lead (II) ions, and temperature on the adsorption process were investigated. Energy Dispersive X-Ray Spectroscopy (EDS) analysis after adsorption reveals the accumulation of lead (II) ions onto activated carbon. The Langmuir and Freundlich isotherm models were applied to analyze equilibrium data. The maximum monolayer adsorption capacity of activated carbon was found to be 476.2 mg g^−1^. The kinetic data were evaluated and the pseudo-second-order equation provided the best correlation. Thermodynamic parameters suggest that the adsorption process is endothermic and spontaneous.

## 1. Introduction

The use of various adsorbents for the removal of heavy metal ions from aqueous solution is of great interest due to environmental concerns. The ground eggshell waste was found as an effective adsorbent for removal of anionic dye from aqueous solution [1]. The removal of cadmium using citrus fruits, apples, and grapes has been investigated [2]. It was reported that citrus peels showed the high adsorption capacity [2]. Activated carbons are widely used for the removal of heavy metal ions from aqueous solution [3–5]. The preparation of granular activated carbon (GAC) from agricultural byproducts and their use in adsorption experiments were reported by Johns et al. [3]. It was concluded that GACs produced from agricultural byproducts were more effective than commercial GACs in terms of adsorption capacity [3]. The removal of organic mercury from the wastewater has been tested using activated carbons and with an ion-exchange resins (Amberlite GT73) [5]. It was reported that activated carbons showed higher adsorption capacity than the ion-exchange resin [5].

There is understandably a great effort to find low cost material to produce the activated carbon. Within the current paper, we describe our efforts to remove lead (II) ions from aqueous solution by using the activated carbon produced from soybean oil cake with chemical activation. Soybean oil cake, an agricultural byproduct, was used for the preparation of the activated carbon. The adsorption of lead (II) ions onto the activated carbon was investigated with variations in the parameters of pH, contact time, lead (II) ions concentration and temperature. The kinetic model for lead (II) adsorption onto the activated carbon was also studied. 

## 2. Experimental 

### 2.1. Materials

The biomass (soybean oil cake) was obtained from Altinyag Oil Company, Izmir, Turkey. The sample contained 17.86 wt% extractives, 52.51 wt% hemicellulose, 2.80 wt% lignin, and 21.58 wt% cellulose. The elemental analysis of the soybean oil cake is as follows: 44.48 wt% C, 6.28 wt% H, 8.21 wt% N, 0.54 wt% S, 40.49 wt% O (by difference), and 5.83 wt% ash content. All chemicals used in the present study were of analytical grade.

### 2.2. Preparation of the Activated Carbon

Preparation of the activated carbon from soybean oil cake by K_2_CO_3_ activation with the impregnation ratio of 1.0 was carried out. K_2_CO_3_ was mixed with the soybean oil cake overnight so that reagents were fully absorbed into the biomass. The slurry was then dried at 105°C. The impregnate material was set in a reactor and then it was carbonized at 1073.15 K. The experimental details for the preparation of activated carbon can be found in a previous report [6]. The yield of the activated carbon was found to be 11.56 wt%. The activated carbon, designated as SAC2, was sieved to particles <63 *μ*m size and used for experiments. A measurement of specific surface areas of the activated carbon produced from soybean oil cake by chemical activation with K_2_CO_3_ has been made by N_2_ adsorption (at 77 K), using a surface analyzer (Quantachrome Inst., Nova 2200e). Surface charge distribution of SAC2 was measured as a function of pH by using a Malvern Zetasizer Nanoseries. The elemental compositions of the activated carbon were determined using a LECO CHNS 932 Elemental Analyzer. The physicochemical properties of the activated carbon are as follows: 81.03 wt% C, 0.53 wt% H, 0.06 wt% N, 0.05 wt% S, 18.33 wt% O (by difference); 0.98 wt% ash content, 1352.86 m^2^ g^−1^ specific surface area, 0.680 cm^3^ g^−1^ total pore volume, 0.400 cm^3^ g^−1^ micropore volume, and 10.05 Å average pore diameter. 

### 2.3. Adsorption Experiments

The adsorption experiments were done in a batch system. Certain amount of SAC2 was added to a lead (II) nitrate solution in an Erlenmeyer flask closed with a glass stopper and the flask content stirred using a magnetic stirrer at 200 rpm to determine the optimum values of pH, initial concentration of lead (II) ions. 

A stock solution containing 1000 mg L^−1^ of lead (II) ions was used for the adsorption experiments. The required lead (II) concentrations were provided with the dilution using deionized water. 100 mL of a lead (II) solution containing 50 mg of the adsorbent in a 250 mL stopper conical flask was agitated at 200 rpm in a water bath, of which temperature was controlled at desired temperature (298.15, 308.15, and 318.15 K). The lead (II) ions concentration of the solution was determined by atomic absorption spectrometry (Perkin Elmer A. Analyst 800 Model). The amount of lead (II) ions on the adsorbent at equilibrium was determined from the difference between the initial and final concentrations of the lead (II) solutions.

SAC2 after adsorption of lead (II) ions was dried in an oven under vacuum at 50°C for 24 h, and then the lead (II) ions adsorbed SAC2 were characterized by Field Emission Scanning Electron Microscope (SEM, Carl Zeiss Ultra Plus) equipped with Energy Dispersive X-ray Spectrophotometer (EDS) analysis.

## 3. Results and Discussion

### 3.1. Effect of pH

**Figure 1:**
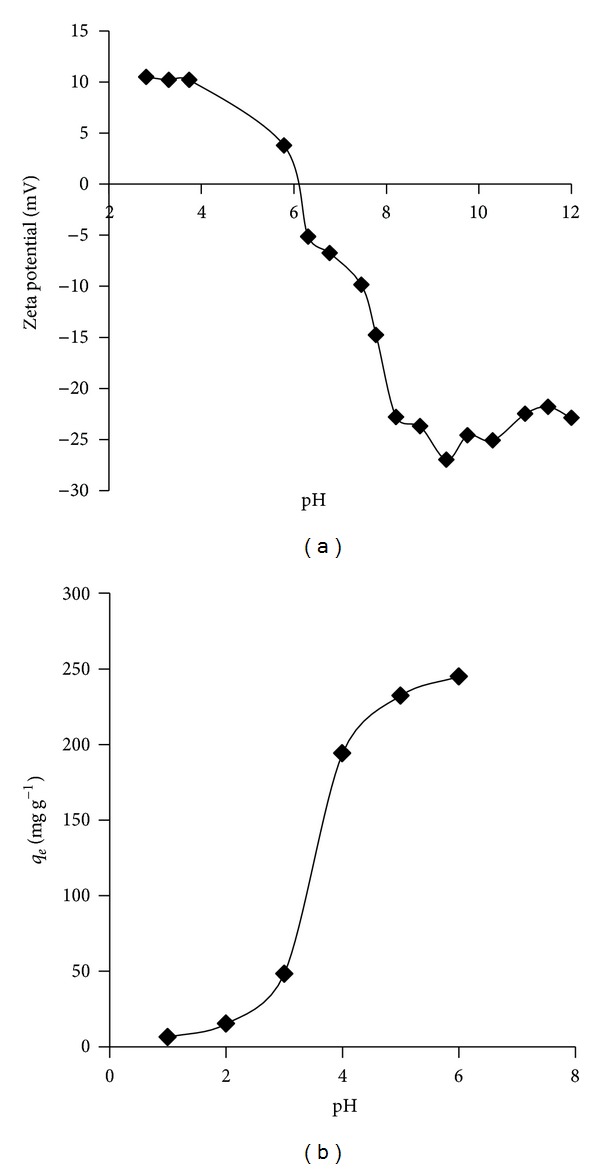
(a) Zeta potentials of SAC2 as a function of pH, (b) effect of pH for the adsorption of lead (II) ions onto the activated carbon (SAC2). (*C*_*o*_ = 300 mg L^−1^; *m* = 50 mg; *V* = 100 mL; *T* = 25°C; agitation rate 200 rpm).

The effect of pH on the lead (II) ion adsorption capacity of SAC2 was studied at 300 mg L^−1^ initial lead (II) ion concentration and at 298.15 K. The pH of solutions is a factor which plays an important role in the adsorption process. Because lead (II) ions precipitate as lead (II) hydroxide at pH values higher than 6.7 [15], above this pH value adsorption experiments were not carried out. The amphoteric nature of carbon has affected both the surface functional groups and the point of zero charge (pHPZC) of the activated carbon [16, 17]. Cationic adsorption is favored at pH > pHPZC and anionic adsorption is favored at pH < pHPZC. Zeta potentials and adsorption capacity of SAC2 regarding the solution pH are illustrated in Figures 1(a) and 1(b), respectively. As can be seen from the figure, pHPZC of SAC2 is 6,1 and the surface was positively charged when the solution pH was below the pH of 6,1. The magnitude of the surface charge of SAC2 was reduced while the pH was increased from 2 to 6. Increased positive charge density on the sites of the activated carbon surface at low pH values (less than 3) blocked to come close of metal cations. On the contrary, when the pH value increased, the electrostatic repulsion between lead (II) ions was decreased and the surface of SAC2 became less positively charged, and the adsorption capacity of SAC2 increased. Maximum adsorption capacity was found as 244.9 mg g^−1^ at pH 6.0. 

### 3.2. Effect of Contact Time

**Figure 2:**
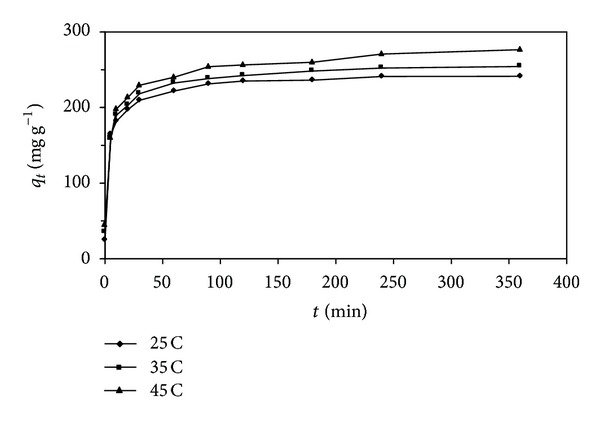
Effect of contact time for the adsorption of lead (II) ions onto the activated carbon (SAC2). (*C*_*o*_ = 300 mg L^−1^; *m* = 50 mg; *V* = 100 mL; pH = 5.5; agitation rate 200 rpm).

A series of contact time experiments for the adsorption of lead (II) ions onto SAC2 were carried out at the initial concentration of lead (II) ions (300 mg L^−1^) and temperatures of 298.15, 308.15, and 318.15 K. The effects of contact time on the adsorption process are shown in Figure 2. The adsorbed amount of lead (II) ions was increased with an increase in contact time up to 100 min, after that there was no significant increase in the adsorption of lead (II) ions onto SAC2. At a 60 min of contact time, the adsorbed amounts of lead (II) ions onto SAC2 were 221.9, 232.6, and 240.2 mg g^−1^ at 298.15, 308.15, and 318.15 K, respectively.

### 3.3. Effect of Initial Lead (II) Ions Concentration

**Figure 3:**
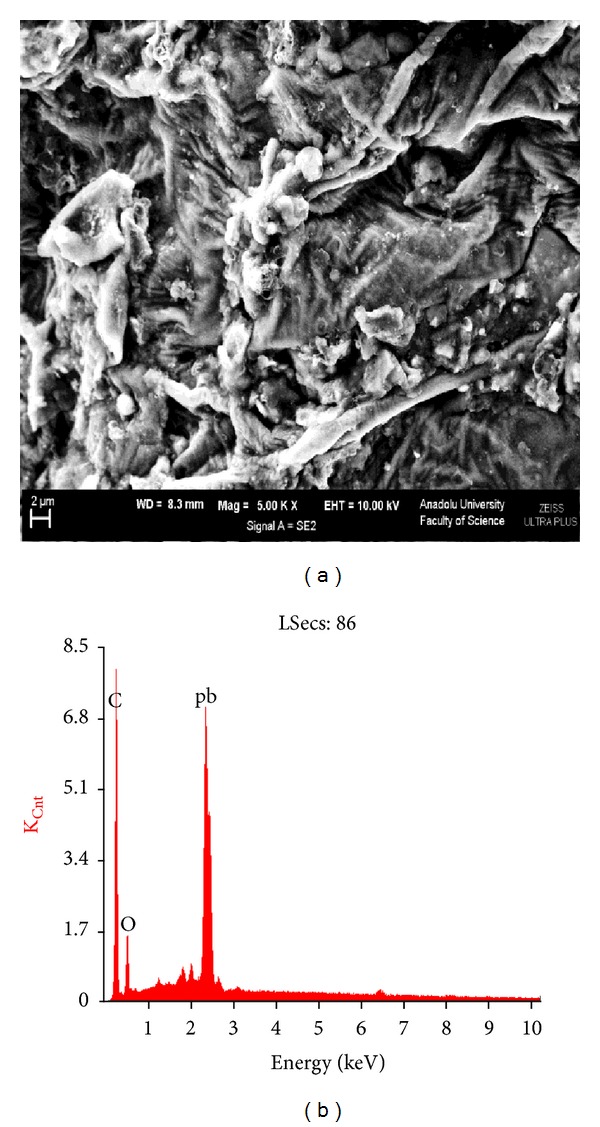
SEM image and EDS spectrum of SAC2 after lead (II) adsorption.

The adsorption capacity of SAC2 for lead (II) ions was increased with an increase in the initial lead (II) ion concentration. Increases in the initial concentration of lead (II) ions cause the mass transfer from the aqueous phase to the solid phase. The maximum adsorption capacities were obtained at the initial lead (II) ion concentration of 500 mg L^−1^. The SEM image and X-ray spectrum of SAC2 after adsorption can be seen in Figure 3. The existence of a peak on the spectrum belonging to lead clearly proves that the accumulation of lead (II) ions onto SAC2 occurred.

### 3.4. Adsorption Kinetics

To investigate the adsorption process of lead (II) ions onto SAC2, the pseudo-first-order kinetic [18], pseudo-second-order kinetic [19], and intraparticle diffusion models [20] were applied to the experimental data.

The pseudo-first-order kinetic model equation is shown as

(1) $$\begin{array}{c} \ln\left(q_{1}-q_{t}\right)=\ln q_{1}-k_{1}t, \end{array}$$

where *q*_1_ and *q*_*t*_ are the amounts of lead (II) ions (mg g^−1^) absorbed at equilibrium and at time *t*, respectively, and *k*_1_ is the first-order rate constant (min^−1^). 

The pseudo-second-order kinetic model is shown as

(2) $$\begin{array}{c} \frac{t}{q_{t}}=\frac{1}{k_{2}q_{2}^{2}}+\frac{1}{q_{2}}t, \end{array}$$

where *q*_2_ is the maximum adsorption capacity (mg g^−1^) for the pseudo-second-order adsorption and *k*_2_ is the equilibrium rate constant for the pseudo-second-order adsorption (g mg^−1^ min^−1^). 

The intraparticle diffusion can be presented by the following equation:

(3) $$\begin{array}{c} q_{t}=k_{p}t^{1/2}+C, \end{array}$$

where *C* is the intercept and *k*_*p*_ is the intraparticle diffusion rate constant (mg g^−1^ min^−1/2^).

**Figure 4:**
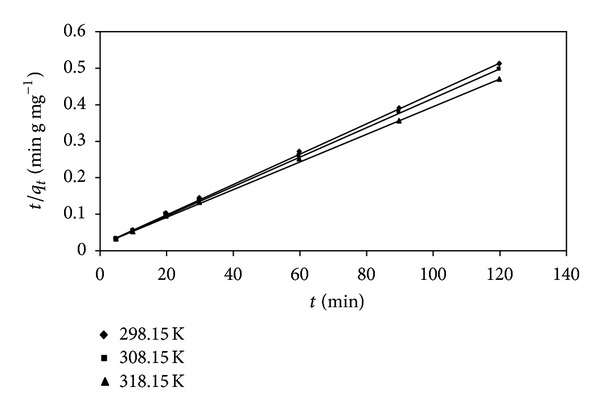
Pseudo-second-order kinetic plot for the adsorption of lead (II) ions onto the activated carbon (SAC2).

The plots of linear form of the pseudo-first-order (not shown), pseudo-second-order, and intraparticle diffusion (not shown) for the adsorption of lead (II) ions onto SAC2 were obtained at the temperatures of 298.15, 308.15, and 318.15 K. The results of kinetic parameters are shown in Table 1. The values of the correlation coefficients of the pseudo-second-order kinetic model (*r*_2_^2^) were higher than those of the *r*_1_^2^ of the pseudo-first-order kinetic model and *r*_*p*_^2^ of the intraparticle diffusion model. This indicates that the adsorption of lead (II) ions followed the pseudo-second-order kinetic with the correlation coefficients of higher than 0.99 for all tested temperatures.  Figure 4 gives the plots of *t*/*q*_*t*_ versus *t* for the adsorption process at different temperatures. With increasing the temperature, the values of the correlation coefficients of the pseudo-first-order kinetic model decreased. 

### 3.5. Adsorption Thermodynamics

Thermodynamic parameters consisting of Gibbs free energy change (Δ*G*°), enthalpy change (Δ*H*°), and entropy change (Δ*S*°) were calculated from the following equation:

(4) $$\begin{array}{c} \Delta G^{{}^{\circ}}=-RT\ln K_{L}, \end{array}$$

where *R* is the universal gas constant (8.314 J mol^−1^ K^−1^), *T* is the temperature (K), and *K*_*L*_ value was calculated using the following equation:

(5) $$\begin{array}{c} K_{L}=\frac{q_{e}}{C_{e}}, \end{array}$$

where *q*_*e*_ and *C*_*e*_ are the equilibrium concentration of lead (II) ions onto the activated carbon (mg g^−1^) and in the solution (mg L^−1^), respectively. 

The enthalpy change (Δ*H*°) and entropy change (Δ*S*°) of the adsorption were estimated from the following equation:

(6) $$\begin{array}{c} \ln K_{L}=\frac{\Delta S^{{}^{\circ}}}{R}-\frac{\Delta H^{{}^{\circ}}}{RT}. \end{array}$$

The enthalpy change (Δ*H*°) and entropy change (Δ*S*°) can be obtained from the slope and intercept of a Van't Hoff equation of (Δ*G*°) as follows:

(7) $$\begin{array}{c} \Delta G^{{}^{\circ}}=\Delta H^{{}^{\circ}}-T\Delta S^{{}^{\circ}}, \end{array}$$

where Δ*G*° is the Gibbs free energy change (J), *R* is the universal gas constant (8.314 J mol^−1^ K^−1^), and *T* is the absolute temperature (K). 

**Figure 5:**
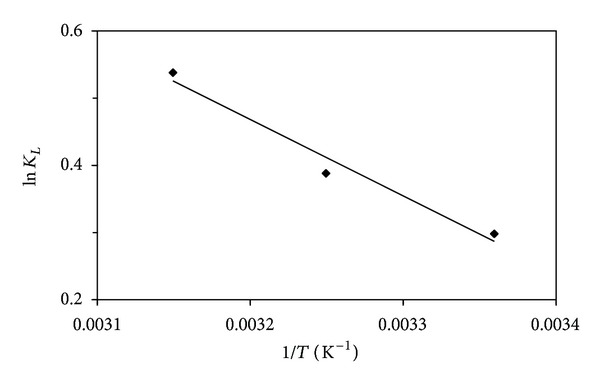
Plot of ln⁡⁡*K*_*L*_ versus 1/*T* for estimation of thermodynamic parameters for the adsorption of lead (II) ions onto the activated carbon (SAC2).

Thermodynamic parameters are listed in Table 2. The Gibbs free energy change (Δ*G*°) is an indicator of the degree of the spontaneity in the adsorption process. In order to provide a better adsorption, it is necessary to have a negative value for the Gibbs free energy changes (Δ*G*°). The values of Gibbs free energy change (Δ*G*°) of lead (II) ions adsorption were determined as 0.74, −0.99, and −1.40 kJ mol^−1^ at the temperatures of 298.73, 308.73, and 318.73 K, respectively. These values indicate that the adsorption process is spontaneous and feasible under these conditions. The values of Δ*G*° at higher temperature are more negative than those of lower temperature. This means that high efficiency of adsorption takes place at high temperatures [21]. Plot of ln⁡⁡*K*_*L*_ versus 1/*T* for estimation of thermodynamic parameters for the adsorption of lead (II) ions onto SAC2 is shown in Figure 5. The positive value of Δ*S*° reflects an increase in the degree of freedom of the adsorbent surface. Similar observation was reported in the literature [22]. The positive value of Δ*H*° for the adsorption of lead (II) onto SAC2 suggests an endothermic nature of process.

**Figure 6:**
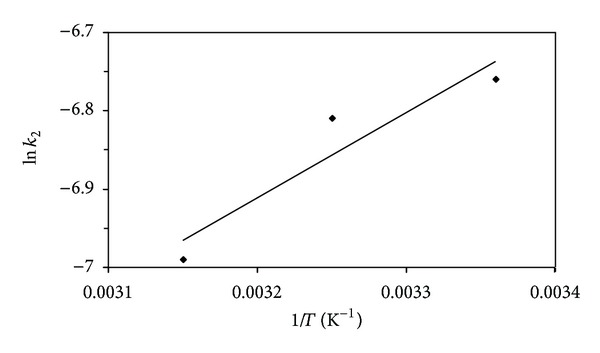
Plot of ln⁡⁡*k*_2_ versus 1/*T* for estimation of activation energy for the adsorption of lead (II) ions onto the activated carbon (SAC2).

Plot of ln⁡⁡*k*_2_ versus 1/*T* for estimation of activation energy for the adsorption of lead (II) ions onto SAC2 is presented in Figure 6. Activation energy was found to be 9.02 kJ mol^−1^ at 308.73 K. 

### 3.6. Adsorption Isotherms

The adsorption data was analyzed with the use of Langmuir and Freundlich isotherms [23, 24].

Langmuir isotherm:

(8) $$\begin{array}{c} \frac{C_{e}}{q_{e}}=\frac{1}{q_{\max}K_{L}}+\frac{C_{e}}{q_{\max}}, \end{array}$$

where *q*_*e*_ is the equilibrium lead (II) ions concentration on the activated carbon (mg g^−1^), *C*_*e*_ is the equilibrium lead (II) ions concentration in the solution (mg L^−1^), *q*_max⁡_ is the monolayer adsorption capacity of activated carbon (mg g^−1^), and *K*_*L*_ is the Langmuir adsorption constant (L mg^−1^). 

**Figure 7:**
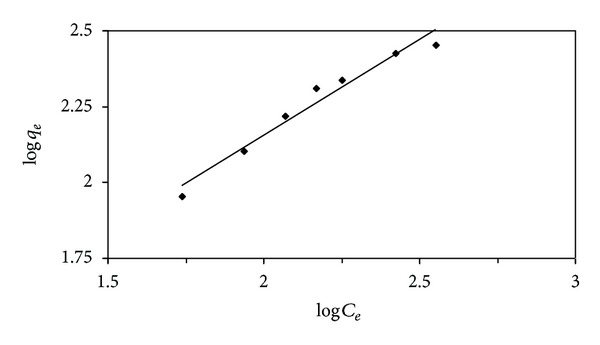
Freundlich plot for the adsorption of lead (II) ions onto the activated carbon (SAC2) at 298.15 K.

Freundlich isotherm:

(9) $$\begin{array}{c} \log q_{e}=\log K_{F}+\frac{1}{n}\log C_{e}, \end{array}$$

where *q*_*e*_ is the equilibrium lead (II) ions concentration on the activated carbon (mg g^−1^), *C*_*e*_ is the equilibrium lead (II) ions concentration in the solution (mg L^−1^), and *K*_*F*_ (L g^−1^) and *n* are the Freundlich adsorption isotherm constants. The plots of log⁡⁡*q*_*e*_ versus log⁡⁡*C*_*e*_ for the adsorption of lead (II) ions onto the activated carbon are shown in Figure 7. The Langmuir and Freundlich isotherm parameters are given in Table 3. The *r* ^2^ value of the Freundlich model is higher than that of the Langmuir model. This shows that the Freundlich model fits better than the Langmuir model. The Freundlich isotherm model suggests heterogeneous surface [25]. A comparison for lead (II) ion adsorption capacities of activated carbons produced from various lignocellulosic materials is tabulated in Table 4 [3, 7–14]. The maximum monolayer adsorption capacity of SAC2 from Langmuir isotherms for lead (II) ions is found to be the highest in comparison with the literature [3, 7–14]. 

## 4. Conclusions

Removal of heavy metal ions from aqueous solution by the activated carbon produced from soybean oil cake has been carried out successfully. The main conclusions are as follows.

- The adsorption capacity for lead (II) ions was increased with an increase in the initial concentration of lead (II) ions.
- The kinetic modeling of the process followed the pseudo-second-order kinetic model at all tested temperatures.
- The adsorption process fitted the Freundlich model.
- The maximum monolayer adsorption capacity of the activated carbon was 476.2 mg g^−1^ which is quite high in comparison with the values in the literature.

Consequently, conversion of a byproduct from the vegetable oil industry to the activated carbon and its use on the adsorption of lead (II) ions from aqueous solution are very important from the viewpoint of economic and environmental aspects. 

## Figures and Tables

**Table 1:** Kinetic parameters for the adsorption of lead (II) ions onto the activated carbon (SAC2).

Temperature (K)	298.15	308.15	318.15
Pseudo-first-order			
*k*_1_ (min^−1^)	0.023	0.020	0.017
*q*_1_ (mg g^−1^)	73.55	79.84	92.29
*r*_1_^2^	0.980	0.928	0.921
Pseudo-second-order			
*k*_2_ (g mg^−1^ min^−1^)	11.6 × 10^−4^	10.96 × 10^−4^	9.26 × 10^−4^
*q*_2_ (mg g^−1^)	238.1	250.0	263.2
*r*_2_^2^	0.9995	0.9993	0.9994
Intraparticle diffusion			
*k*_*p*_ (mg g min^−1/2^)	8.7429	9.9775	11.319
*C*	153.74	152.85	154.53
*r*_*p*_^2^	0.9390	0.8791	0.8659

**Table 2:** Thermodynamic parameters calculated from the Langmuir isotherm constant, *K*_*L*_, and activation energy calculated from the pseudo-second-order rate equation, *k*_2_, for the adsorption of lead (II) ions onto the activated carbon (SAC2).

*T* (K)	*E*_*a*_ (kJ mol^−1^)	Δ*G*° (kJ mol^−1^)	Δ*H*° (kJ mol^−1^)	Δ*S*° (J K^−1^ mol^−1^)
298.15		−0.74		
308.15	9.02	−0.99	9.46	34.15
318.15		−1.40		

**Table 3:** Adsorption isotherms constants for the adsorption of lead (II) ions onto the activated carbon (SAC2) at 298.15 K.

Langmuir	
*q*_ max_ (mg g^−1^)	476.19
*K*_*L*_ (L mg^−1^)	2.201
*R*_*L*_	0.419
*r*_*L*_^2^	0.9413

Freundlich	

*n*	1.586
*K*_*F*_ (L g^−1^)	7.381
*r*_*F*_^2^	0.9624

**Table 4:** Comparison of adsorption capacities of activated carbons obtained from various lignocellulosic materials for lead (II) ions.

Biomass	pH	*T* (K)	Operating conditions		
			Initial concentration or range (mg L^−1^)	Amount of adsorbent (g L^−1^)	Adsorption capacity (mg g^−1^)
Apricot stone [7]	6.5	298.15	—	2.0	22.85
Soybean hulls [3]	5.0	296.15	518	10.0	39.37
Pecan shell [8]	4.8	—	104	0.5–10.0	64.2
Coconut shell [9]	5.6	298.15	—	2.0	76.66
Palm shell [10]	3.0 and 5.0	300.15	100–700	5.0	95.20
Sawdust [11]	5.0	300.15	50–1000	2.0	200.00
Bagasse pith [12]	4.0–8.0	303.15	100	—	200.00
Coir pith waste [13]	4.0	—	25–100	—	263.00
Euphorbia rigida [14]	5.0	313.15	50–200	0.8	279.72
Soybean oil cake*	5.5	298.15	50–500	0.5	476.2

*Present study.
